# Antihyperglycemic, antihyperlipidemic and antioxidant activity of phenolic rich extract of *Brassica oleraceae var gongylodes* on streptozotocin induced Wistar rats

**DOI:** 10.1186/s40064-015-0948-0

**Published:** 2015-05-03

**Authors:** Indumati Sharma, Mallikarjun Aaradhya, Madhuri Kodikonda, Prakash Ramchandra Naik

**Affiliations:** DOS in Zoology, University of Mysore, Manasagangothri, Mysore, 570006 India; Fruit and Vegetable Technology, Central Food Technological Research Institute, Mysore, 570020 India

**Keywords:** STZ, *Brassica oleraceae var gongylodes*, Hypoglycemic, Hypolipidemic, Antioxidant

## Abstract

Cruciferous vegetables, in particular those included into the Brassica genus, are good sources of a variety of nutrients and health-promoting phytochemicals. Phenolic compounds are the major antioxidants of Brassica; hence the contribution of Brassica vegetables to health improvement has largely been associated to their antioxidant capacity. This study aimed to assess anti-diabetic, antilipidemic, and antioxidant activity of phenolic rich extract of *Brassica oleraceae var gongylodes (BOvG)* in Wistar rats. The findings revealed that the administration of BOvG extract to diabetic rats significantly reduced fasting blood glucose by 64% within 7 days of treatment. Additionally, BOvG extract was also observed to normalize the diabetic rats’ lipid profile and HbA1c (Glycated hemoglobin). BOvG extract also showed protection of liver- kidney functions, which was evidenced by the significant decrease in Blood Urea Nitrogen (BUN), Serum glutamic oxaloacetic transaminase (SGOT) and Serum glutamic pyruvic transaminase (SGPT). The treatment also improved the antioxidant status of the diabetic rats where the enzymatic activities of Catalase (CAT) and Super Oxide Dismutase (SOD) were significantly increased. Furthermore, RP-HPLC analysis detected chlorogenic acid, rutin, and sinapic acid against known standards in BOvG extract. Hence, the present investigation suggests that BOvG phenolic rich extract (as a multi-component therapy) exhibited anti-diabetic, antilipidemic and antioxidant properties in STZ-induced diabetic rats.

## Introduction

In the last few decades, there has been a tremendous increase in the prevalence of type 2 diabetes internationally, and has been estimated that the number of diabetic patients will be more than 205 million in the next 20 years (Diabetes Atlas and 6th Edition 2014). Oral hypoglycemic agents and insulin, generally used to treat Diabetes Mellitus (DM), have number of serious side effects (Kyriacou and Ahmed 2010). Hence, interest has increased in finding naturally occurring anti-diabetic therapeutics to replace synthetic drugs (Velioglu et al. 1998). As there have been increasing emphasis on the promotion of a healthy diet for the management of type 2 diabetes, there is a great necessity to investigate the combination of drugs derived from natural resource extracts (Kaur et al. 2013).

Numerous studies have established an inverse correlation between the intake of fruits and vegetables and the onset of diseases such as diabetes, cardiovascular disease, cancer, and aging-related disorders as they are rich source of dietary antioxidants, including polyphenolics, vitamins E and C, and carotenoids (Huang et al. 2005). Polyphenolic compounds, in particular, have been shown to terminate free radical chain reactions in biological systems, and hence can act as nutraceuticals for a range of oxidative stress implicated diseases, like diabetes and cancer (Espín et al. 2007).

Amongst plant foods with health benefits, crops from the family Brassicaceae (also known as Cruciferae) have been the focus of numerous epidemiological and clinical studies (Podsędek 2007) as they are good source of variety of nutrients and health promoting phytochemicals (Liu 2004; Soengas et al. 2011). Brassica vegetables, like knol khol, cabbage, broccoli, cauliflower, kale and Brussels sprouts are widely consumed throughout the world. Folklore medicine have shown that knolkhol/kohlrabi, scientifically known as *Brassica oleraceae var gongylodes* (BOvG) has health promoting activity and anti-diabetic activity. Previously (Huchaiah et al. 2008) BOvG juice was assessed for hypoglycemic activity and its related biochemical parameters. However, till date phenolic rich extract of BOvG and its components have not been investigated and hence the present investigation assessed anti-diabetic activity of phenolic rich extract of BOvG.

## Methodology

### Induction of diabetes in rats

Wistar rats weighing 160 ± 15 g were housed in an animal facility. The animals were acclimatized to the environment (26 ± 5°C, 55 ± 10% relative humidity, and 12 h dark/light cycle) for 1 week prior to experimental use. The rats were fed with a standard laboratory diet (Amruth feeds Pvt. Ltd, Bangalore) and water ad libitum. Ethical clearance was obtained from Institutional Animal Ethics Committee of Animal Research (IAEC approval number: UOM/IAEC/09/2012) at DOS in Zoology, University of Mysore, and experiments were carried out as per the guidelines of the committee.

The animals were divided into 4 groups and each group consisted of 5 rats:

- Group 1: Control- normal untreated rats (citrate buffer injected)
- Group 2: Diabetic- STZ diabetic rats
- Group 3: BOvG_800_- STZ rats treated with BOvG (800 mg/kg body weight)
- Group 4: Glibenclamide- STZ rats treated with glibenclamide (1 mg/kg body weight)

Overnight fasted animals were made diabetic by intra-peritoneal injection of freshly prepared streptozotocin in citrate buffer (0.1 M, pH 4.5) at a dose of 47 mg/kg body weight. The control rats were only injected with citrate buffer. After 96 h of induction when blood glucose was stabilized, fasting blood glucose (FBG) was determined and rats having FBG >250 mg/dl were designated as having diabetes mellitus and were used in this experiment. The experimental period lasted for 28 days and day 0 was designated as the day when rats were confirmed to be diabetic.

STZ untreated rats were left as such for the entire experimental duration. BOvG extract was dissolved in distilled water and was given orally once daily using an intra-gastric gavage for 28 days for diabetic rats. Glibenclamide (1 mg/body weight) was administered as positive control.

### Preparation of plant extract

The knob of BOvG was washed under running water thoroughly, chopped into slices, dried in a hot air oven at 45°C for 24 h, and powdered to 60 mesh in an apex grinder. The powdered sample was then extracted serially using solvents with increasing polarity, namely, hexane, ethyl acetate, and double distilled water at room temperature (25°C), so as to extract both non-polar and polar bioactive compounds. The extraction process was repeated until the solvent became colorless. The extracts were then filtered (Whatman No.1 filter paper) and dried using a rotary evaporator (Hahnvapor, model no. HS-2005S, Korea). The solvents in the poly phenolic rich extract (or the aqueous extract) were allowed to evaporate completely in a hot air oven at 32°C and the extract was stored there until further use.

### Determination of phenolic compounds

Analytical HPLC was carried out to determine the presence of phenolic compounds in the BOvG extract (Francisco et al. 2010). Briefly, the analysis was carried out on a Luna C18; column (250 mm × 4.6 mm, 5 μm particle size; Phenomenex, Macclesfield, UK). The mobile phase was a mixture of: (A) HPLC grade water/trifluoro acetic acid (TFA) (99.9:0.1) and (B) methanol/TFA (99.9:0.1). The flow rate was 1 mL min-1 in a linear gradient starting with 0% B at 0–5 mins, reaching 17% B at 15–17 min, 25% B at 22 mins, 35% B at 30 mins, 50% B at 35 mins, 99% B at 50 mins and at 55–65 mins 0% B. The injection volume was 20 μl and chromatograms were recorded at 320 nm for phenolic derivatives. Polyphenolics were identified by sinapic acid (Sigma-Aldrich), chlorogenic acid (Sisco Research Laboratories Pvt. Ltd) and rutin (Sigma-Aldrich,) as standards.

### Fasting Blood Glucose (FBG)

The animals were fasted overnight and the blood from tail vein was estimated for fasting blood glucose by glucometer (from Accu-Chek) in which its measurement principle is based on glucose dehydrogenase method. The blood glucose was measured on days 0, 7, 14, 21 and 28 days after treatment.

### Biochemical parameters

All rats were anesthetized by diethyl ether after recording the final body weight. The blood samples of each animal were collected through cardiac puncture and serum was obtained by centrifuging blood samples at 4000 rpm at 25°C for 4 min and analyzed for assorted biochemical parameters. The obtained serum samples was preserved at −20°C. The total cholesterol (TC), high density lipoprotein (HDL) cholesterol, low density lipoprotein (LDL) cholesterol, total triglyceride (TG), SGOT, SGPT and BUN was done through Artos - Versatile Clinical Chemistry Analyzer using standard kits (supplied by Swemed biomedicals pvt. ltd., India). Whole blood was collected separately (preserved with anticoagulating agents) for the estimation of glycosylated hemoglobin using standard kits (supplied by Swemed biomedicals pvt. ltd., India).

### Estimation of antioxidant enzymes

Antioxidant enzyme was estimated by liver and kidney homogenate (10% homogenate), prepared in chilled phosphate buffer solution of pH 7.0. This was used to measure the levels and activities of superoxide dismutase (Marklund and Marklund 1974) and catalase (Sinha 1972) through standard protocols. SOD was assayed by autoxidation of pyrogallol while CAT was assayed by decomposition of hydrogen peroxide. The activities were expressed as unit/mg of protein (a unit is defined as the amount which will catalyse the transformation of 1 micromole of substrate (or product) per min, under defined assay conditions).

### Statistical analysis

All the data were expressed as the mean ± SEM and analysis of variance (ANOVA) was used for the statistical analysis using SPSS 11.5 followed by Duncan Multiple Range post hoc test. The values were considered to be significant when p < 0.05.

## Results

### Effect of BOvG on body weight and on Fasting Blood Glucose (FBG) level

**Figure 1:**
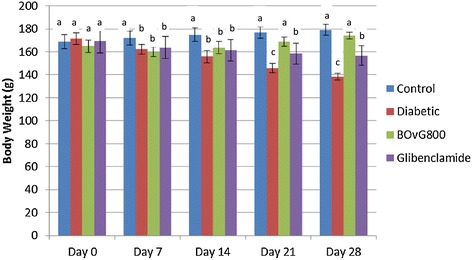
Effect of BOvG extract on body weight.

Body weight of control groups increased over a period of time and there was not much difference in weight gain amongst the control groups at the end of the experimental period (Figure 1). On the other hand there was loss of body weight (by 24%) in the diabetic group. Upon treatment with BOvG extract, BOvG_800_ group’s body weight was similar to that of control group and was significantly different (P < 0.05) from diabetic group. Glibenclamide group showed increase in body weight (significantly different from diabetic group, P < 0.05) but it did not equal that of control (P < 0.05).

**Figure 2:**
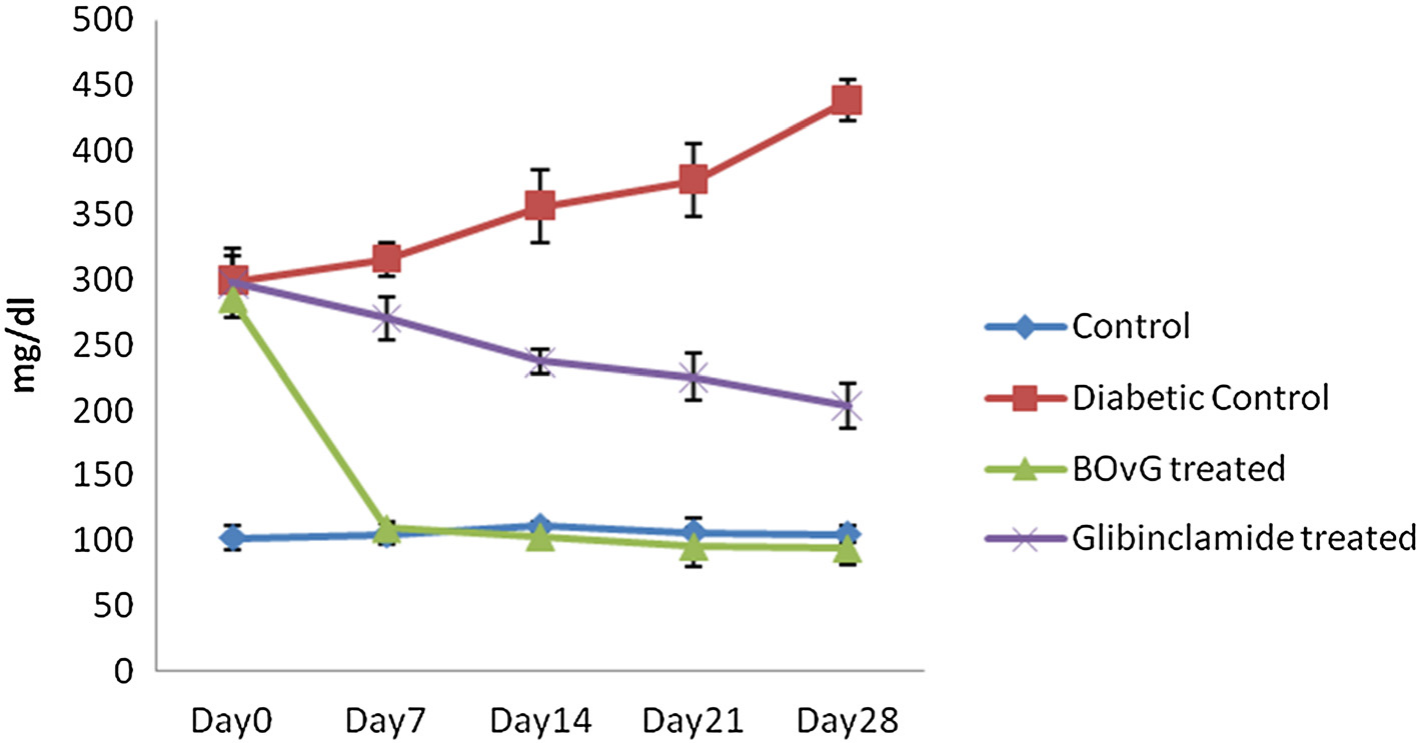
Effect of BOvG extract on plasma glucose levels.

The fasting blood sugar (FBS) of control groups over the course of 4 weeks was 99–113 mg/dl and was not significantly (P > 0.05) different from one another (Figure 2). Diabetic groups showed steep increase in FBS and at the end of 4th week the FBS was 420 mg/dl. Treatment with BOvG extract, however, reduced the FBS in the first week to 98.5 mg/dl (by 64%) and was maintained as such for the entire experimental period. BOvG group showed no significant difference (P < 0.05) compared to control from week 1 to the end of week 4. Similarly glibenclamide group also showed reduction in FBS to 180 mg/dl (by 42%) at the end of 4th week. BOvG extract showed more potency for anti-hyperglycemic activity than glibenclamide.

### Effect of BOvG on lipid profile

To evaluate the effect of BOvG extract on lipid profile level, TC (total cholesterol), TG (triglyceride), LDL (low density lipoprotein) and HDL (high density lipoprotein) were assessed at the end of 4 weeks. Diabetic rats showed an increase of 47.5% in LDL, 27% in TC, 46% in TG and a 61% decrease in HDL (Table 1). Diabetic rats were significantly different from control (P < 0.05). BOvG_800_ group, contrarily, showed restoration of LDL, HDL, TC and TG to the levels of control rats as there was no significant difference between BOvG_800_ group and control group. In the glibenclamide group LDL, HDL and TG showed marginal restoration of lipid profile levels as it was still significantly different to that of control group (P < 0.05). However, TC of glibenclamide group showed full restoration of the normal levels as it was not significantly different from control.

**Table 1:** **Effect of BOvG on lipid profile**

**Group**	**LDL (mg/dL)**	**HDL (mg/dL)**	**TC (mg/dL)**	**TG (mg/dL)**
Control	42.60 ± 2.9^a^	41.20 ± 2.8^a^	73.80 ± 3.1^a^	81.3 ± 2.6^a^
Diabetic	80.37 ± 3.5^c^	16.70 ± 2.0^c^	100.50 ± 6.2^b^	152.03 ± 3.0^c^
BOvG_800_	43.35 ± 1.9^a^	38.82 ± 2.4^ba^	74.27 ± 1.6^a^	90.34 ± 3.7^a^
Glibenclamide	51.33 ± 1.3^b^	32.92 ± 2.5^b^	78.97 ± 2.3^a^	115.26 ± 4.1^b^

*Note*. Values are mean ± SEM (n = 5). Means in the same columns with different superscripts differ significantly (p < 0.05).

### Effect of BOvG extract on glycated hemoglobin (HbA1c)

The diabetic rats showed increased levels (32%) of HbA1c and significantly (P < 0.05) differed to that of control group (Table 2). The BOvG_800_ group showed decrease (43%) in HbA1c levels and did not show significant difference to that of control. Glibenclamide group too showed restoration of HbA1c levels compared to control significantly (P < 0.05).

**Table 2:** **Effect of BOvG on serum biochemical markers**

**Group**	**BUN (mg/dl)**	**SGPT (Iu/L)**	**SGOT (Iu/L)**	**HbA1c (%)**
Control	6.98 ± 0.4^bc^	6.90 ± 0.4^bc^	76.36 ± 2.1^c^	76.10 ± 1.5^c^
Diabetic	3.20 ± 0.4^a^	4.01 ± 0.3^a^	50.25 ± 4.6^a^	29.50 ± 2.2^a^
BOvG_800_	7.56 ± 0.5^c^	7.43 ± 0.3^c^	71.25 ± 1.3^bc^	72.00 ± 1.3^c^
Glibenclamide	6.19 ± 0.4^b^	6.28 ± 0.3^b^	66.75 ± 1.7^b^	63.50 ± 0.7^b^

*Note*. Values are mean ± SEM (n = 5). Means in the same columns with different superscripts differ significantly (p < 0.05).

### Effect of BOvG extract on serum biochemical markers: Blood Urea Nitrogen (BUN), SGOT (Serum glutamic oxaloacetic transaminase), SGPT (Serum glutamic pyruvic transaminase)

The serum biomarkers, BUN, SGOT and SGPT, were estimated for the assessment of kidney’s and liver’s physiological function. BUN levels were significantly (*P* < 0.05) increased (54.75%) in diabetic groups (Table 2), when compared to control groups. The BOvG_800_ group were normalised to that of control group’s levels. Glibenclamide group also showed significant reduction in BUN levels compared to diabetic group (P < 0.05).

The hepatic enzymes SGOT’s and SGPT’s levels in serum increased significantly (P < 0.05) in diabetic groups by 47% and 45% respectively, whereas the BOvG_800_ group’s SGOT and SGPT showed a decrease by 45% and 39% respectively. Glibenclamide groups also restored the SGOT and SGPT levels to that of control group significantly (P < 0.05).

### Effect of BOvG on antioxidant enzymes- Superoxide dismutase (SOD) and Catalase (CAT)

In diabetic group SOD and CAT activity was significantly (P < 0.05) decreased (Table 3). Liver’s and Kidneys’ SOD in diabetic group showed significant (P < 0.05) decrease by 54% and by 42% respectively. However the enzymatic activity was restored in BOvG_800_ and glibenclamide groups and was not significantly different from control.

**Table 3:** **Effect of BOvG on antioxidant enzymes in liver and kidney**

**Groups**	**SOD liver (U/mg of protein)**	**SOD kidney (U/mg of protein)**	**CAT liver (U/mg of protein)**	**CAT kidney (U/mg of protein)**
Control	42.35 ± 2.3^a^	34.43 ± 1.4^a^	42.05 ± 1.3^a^	5.50 ± 0.2^a^
Diabetic	93.89 ± 4.9^b^	62.99 ± 2.0^b^	79.20 ± 3.3^b^	8.10 ± 1.1^b^
BOvG_800_	41.26 ± 3.7^a^	39.38 ± 1.2^a^	43.70 ± 1.9^a^	4.59 ± 0.3^a^
Glibenclamide	45.41 ± 4.1^a^	38.08 ± 2.7^a^	53.29 ± 3.6^a^	5.21 ± 0.2^a^

*Note*. Values are mean ± SEM (n = 5). Means in the same columns with different superscripts differ significantly (p < 0.05).

Catalase activity in diabetic group’s kidney was significantly (P < 0.05) reduced (Table 3). It was observed that kidneys CAT activity (reduction by 62%) was reduced twice as that of liver (reduction by 34%) in diabetic groups. Contrarily, CAT activity was restored in BOvG_800_ group where they showed an increase by 21% in liver and 47% in kidneys, was not significantly different from control. Glibenclamide group was also able to restore CAT activity, but was not as potent as BOvG_800_ group where it was significantly different from control in both liver and kidney.

### Detection of polyphenolics

**Figure 3:**
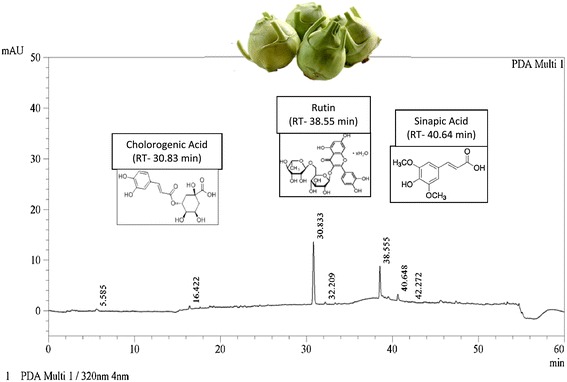
RPHPLC fingerprint chromatogram of BOvG extract and the structures of polyphenols identified.

The major polyphenolic present in BOvG were recognized by comparison to the retention times and absorption spectra of authentic standard markers. Retention times for chlorogenic acid, sinapic acid and rutin were 30.83, 38.55, 40.64 minutes and had concentrations of 5.9 mg/g, 2.7 mg/g and 1.6 mg/g respectively (Figure 3).

## Discussion

Diabetes is a metabolic disorder of carbohydrate, fat and protein, affecting a large number of populations in the world. The pathogenesis of diabetes involving hyperglycemia, dysregulated metabolism and the ensuing diabetes specific micro as well as macro vascular complications have posed an immense challenge for its overall treatment.

Most modern drug discovery has been based on a ‘one-disease–one-target–one-drug' strategy (Ji et al. 2009). A multifactorial disease like diabetes requires a combination of different types of nutraceuticals that target multiple pathways rather than a selective compound which often target a single pathway. This ‘multi-component therapeutics’ is gaining huge popularity within the scientific community (Ji et al. 2009), and it is imperative that the pharmaceutical industry realize its importance to overcome the challenge of ‘more investment, fewer drugs'.

The polyphenols in Brassica vegetables have immense health improvement potential as these polyphenols have high antioxidant activity (Cartea et al. 2011). Rasal et al. (2005) demonstrated the anti-diabetic and anti-oxidant activities of petroleum extract of knol khol in diabetic rats. The phenolic rich extract in the present study fared better than the petroleum extract, as it achieved euglycemia within one week of treatment. In this study*,* we have also identified the presence chlorogenic acid (5.9 mg/g), sinapic acid (2.7 mg/g) and rutin (1.6 mg/g) in BOvG. Earlier, chlorogenic acid and its isomers, neo- and cryptochlorogenic acids have been reported in other Brassica species and have been the important predictors for antioxidative capacity in Brassica varieties (Kaulmann et al. 2014).

The most abundant group of polyphenols in Brassica species are the flavonoids and hydroxycinnamic acids (Cartea et al. 2011). Significant levels of chlorogenic acids have previously been reported in leafy Brassica species, like kale, cabbage and Brussels sprouts. Earlier reports have found flavonoid glycosides, hydroxycinnamic acids as well as sinapic acids and their derivatives to be the most predominant phenolics in Brassica sps. (Cartea et al. 2011). In addition to polyphenolics, isothiocynates (hydrolytic products of glucosinolates; a characteristic compound in Brassica), have been identified in early white vienna cultivar of BOvG with 4-methylthiobutyl being the most predominant isothiocynate (Carlson et al. 1987).

In the present investigation, on treating diabetic rats with BOvG extracts, the FBG levels normalized within one week and fared better than the standard drug glibenclamide. This anti-diabetic activity of BOvG extract was possibly attributed to the additive effect of activation of a number of molecular pathways by the various enriched bioactive components. Earlier reports have shown chlorogenic acid stimulates glucose transport in skeletal muscle via the activation of AMPK (Ong et al. 2012) and sinapic acid activates PLC-PKC signals to enhance the glucose utilization GLUT4 in muscle cells (Cherng et al. 2013). Additionally, chlorogenic acid and rutin have shown cholesterol lowering abilities by up-regulating the Gene Expression of PPAR-α (Wan et al. 2013) and by ameliorating oxidative stress genes (Al-Rejaie et al. 2013) respectively.

There are various studies in which different herbal drug combinations have been beneficial for reducing dosage, side effects and the duration of action (Kaur et al. 2013; Rathera et al. 2013). Herbal drugs also have additive effects; thus showing drastic reduction in FBG levels and amelioration of diabetic related complications in BOvG fed rats in this study. In continuation of this study, the combination index of chlorogenic acid, sinapic acid and rutin (the predominant polyphenols found in BOvG) (Rathera et al. 2013; Chou 2006) and the nature of their interactions, needs to be further investigated in a diabetic model.

BOvG has shown in this study and earlier studies (Huchaiah et al. 2008; Rasal et al. 2005) immense potential as a natural alternative to control diabetes. In the short run, BOvG tuber can be an important part of meal planning for diabetic patients in order to manage their post prandial blood glucose level. Moreover, in the longer run, compounds identified in BOvG have the potential to be developed as natural anti-diabetic drugs.

## Conclusion

Based on our current findings BOvG significantly reduced FBG to normal levels and alleviated diabetes related complications. We suggest that the phytomolecules in BOvG have the potential to form a multi-component drug to target diabetes and its related complications.

### Animal rights

Ethical clearance of the animal experiment had been approved by the Institutional Animal Ethics Committee of Animal Research (IAEC approval number: UOM/IAEC/09/2012) at DOS in Zoology, University of Mysore, and experiments were carried out as per the guidelines of the committee.
